# Differentiating Nonfunctional Paraganglioma of the Bladder from Urothelial Carcinoma of the Bladder: Pitfalls and Breakthroughs

**DOI:** 10.1155/2019/1097149

**Published:** 2019-11-06

**Authors:** Musa Male, Tao Ye, Jin Tao, Zhi-qiang Chen, Ejun Peng

**Affiliations:** ^1^Department of Urology, Tongji Hospital, Tongji Medical College, Huazhong University of Science and Technology, Wuhan 430030, China; ^2^Department of Urology, The First Affiliated Hospital of Zhengzhou University, Zhengzhou 450052, China

## Abstract

**Background:**

Although both nonfunctional paraganglioma of the bladder (NPB) and urothelial carcinoma of the bladder (UCB) are subtypes of bladder tumors, they are entirely different entities with distinct tissue origins and anatomical locations. However, NPB is frequently misdiagnosed as UCB chiefly due to the similarities in their clinical characteristics and cystoscopic features. This study aimed to compare the differences in their clinical characteristics and cystoscopic features.

**Patients and Methods:**

Between April 2007 and September 2017, 14 patients with NPB (NPB group) were retrieved from 2 centers, and 42 patients with new-onset UCB (UCB group) were randomly retrieved. Demographic, symptomatic, imaging, and cystoscopic data of patients in both groups were collected and compared.

**Results:**

NPB group comprised 7 males and 7 females, with a mean age of 43.1 ± 13.6 years. Compared with the UCB group, patients in the NPB group were significantly younger (*p* < 0.001), less likely to be male (*p* < 0.05), and to present with hematuria (*p* < 0.01). However, no significant difference in maximum tumor diameter was observed between the 2 groups (*p*=0.609). Compared with the UCB group, cystoscopically, patients in the NPB group were significantly more likely to present with hypervascularization but less likely to present with hemorrhage, necrosis, calcification, pedunculation, and multilesion (*p* < 0.05). No patients with NPB were clinically diagnosed correctly before cystoscopy. Of the 5 patients who underwent both cystoscopy and biopsy, 4 were diagnosed with NPB, while 1 remained undiagnosed. Of the remaining 9 patients who underwent cystoscopy only, 5 were diagnosed with nonepithelial tumor, and 4 were misdiagnosed with UCB.

**Conclusions:**

Age, sex, and hematuria may provide clues to differentiating NPB from UCB. Differences in cystoscopic features between NPB and UCB are of high diagnostic value. Cystoscopic biopsy should be considered in the preoperative diagnosis of NPB.

## 1. Introduction

Paragangliomas of the bladder are rare tumors originating from the autonomic paraganglion tissues embedded in the muscular layer or the lamina propria [1, 2] instead of the epithelial layer. They are divided into functional and nonfunctional phenotypes. The latter, nonfunctional paragangliomas of the bladder (NPBs), lack typical manifestations of catecholamine syndrome [3] and account for approximately 38.7% of all paragangliomas of the urinary bladder. Unlike functional paragangliomas of the urinary bladder characterized by micturition-induced paroxysmal hypertension, headache, palpitation, sweatiness, and syncope [4], NPBs frequently present with nonspecific painless hematuria or no symptom. Urothelial carcinomas of the bladder (UCBs) are the most common malignant bladder tumors. As opposed to NPBs, UCBs arise from the epithelial layer of the bladder wall, frequently presenting with painless hematuria or occasionally without any symptoms. Thus, symptomatically NPBs extremely mimic UCBs.

Although both NPB and UCB are subtypes of bladder tumors, they are independent entities with distinct tissue origins and anatomical locations. More importantly, they are distinct in surgical approaches, adjuvant therapies, follow-up protocols, and expected prognoses. However, due to the rarity and similar symptoms of NPB to UCB, it is frequently clinically [5] or even pathologically [6–8] misdiagnosed as UCB, potentially leading to severe consequences. Even though cystoscopy is the gold standard for diagnosing general bladder tumors, the cystoscopic differences of NPB from UCB remain not well elucidated.

Therefore, further studies are required to avoid the current frequent misdiagnosis. Herein, a retrospective cohort study was conducted to compare the differences in clinical characteristics and cystoscopic features between NPB and UCB.

## 2. Patients and Methods

### 2.1. Study Population

This study was approved by the institutional review boards of Tongji Hospital of Huazhong University of Science and Technology and the First Affiliated Hospital of Zhengzhou University.

From April 2007 to September 2017, 31 patients pathologically diagnosed with paragangliomas of the bladder were retrieved from the above 2 centers. Then 17 patients were excluded from the NPB group based on their clinical characteristics of functional phenotypes including symptoms of hypercatecholaminemia, elevated catecholamines/metabolites levels, and positive results in functional imaging. Eventually, 14 patients with NPB were enrolled in the NPB group.

From April 2007 to September 2017, 300 patients pathologically diagnosed with UCB were randomly retrieved. After excluding the patients with recurrent UCB and those with carcinoma in situ, 147 patients with new-onset UCB remained. Further, 42 patients with UCB were selected (by random number table) and enrolled in the UCB group.

### 2.2. Study Data

Data on sex, age, and symptoms of patients in the NPB and UCB groups were collected. Moreover, their imaging and cystoscopic data were obtained. In addition, surgical and prognostic data of patients in the NPB group were reviewed.

In this study, diagnostic transurethral resection of bladder tumor (TURBT) was considered as cystoscopy + TURBT. The maximum tumor diameter was measured by computed tomography (CT) or ultrasound. Only the larger or largest tumor diameter was recorded for a patient with multiple lesions. The anatomical type of NPB was also determined by CT or ultrasound.

### 2.3. Statistical Analysis

Differences in continuous variables between the NPB and UCB groups were compared using Student's *t*-test or Wilcoxon rank-sum test. Fisher's exact test or Chi-square test was used to test differences in categorical variables between the 2 groups. The above statistical methods are detailed in the corresponding tables. Statistical analyses were processed using SPSS software (version 24, Chicago, IL, USA). Two-tailed *p* values <0.05 were considered statistically significant.

## 3. Results

### 3.1. Characteristics of Patients in the NPB Group

The detailed data of patients in the NPB group are shown in Table 1. The NPB group comprised 7 males and 7 females, with a mean age of 43.1 ± 13.6 years. Of the patients in the NPB group, 78.6% (11/14) had submucosal tumor and 21.4% (3/14) had intramural tumor. The median maximum diameter of the tumors was 1.9 cm (interquartile range (IQR): 1.0–2.6).

### 3.2. Results of Diagnosis and Misdiagnosis

Prior to cystoscopy, no patients with NPB were clinically diagnosed precisely. Of the 5 patients who underwent cystoscopy and biopsy, 4 were correctly diagnosed, whereas 1 remained undiagnosed due to inadequate tissue from biopsy. Among the remaining 9 patients who underwent cystoscopy only, 5 were diagnosed with nonepithelial tumor, and 4 were misdiagnosed with UCB.

### 3.3. Differences in General Clinical Characteristics

Table 2 shows the statistical analyses in age, sex, hematuria, and maximum tumor diameter between the NPB and UCB groups. Patients in the NPB group were significantly younger (43.1 ± 13.6 years *vs* 59.8 ± 12.0 years, *p* < 0.001) and less likely to be male (50% vs 81.0%, *p* < 0.05) than patients in the UCB group. In addition, patients in the NPB group were significantly less likely to present with hematuria than patients in the UCB group (35.7% *vs* 83.3%, *p* < 0.01). However, no significant difference in maximum tumor diameter was observed between the NPB and UCB groups (1.9 cm (IQR: 1.0–2.6) *vs.* 2.0 cm (IQR: 1.0–3.0), *p*=0.609).

### 3.4. Differences in Cystoscopic Features

Table 3 shows the statistical analyses in cystoscopic features between the NPB and UCB groups. Cystoscopically, patients in the NPB group were significantly more likely to present with hypervascularization (71.4% *vs* 21.4%, *p* < 0.01) than patients in the UCB group. However, patients in the NPB group were significantly less likely to present with hemorrhage (28.6% *vs* 71.4%, *χ*^2^ = 8.1, *p* < 0.01), necrosis (7.1% *vs* 38.1%, *p* < 0.05), calcification (0 *vs* 31.0%, *p* < 0.05), pedunculation (35.7% *vs* 71.4%, *χ*^2^ = 5.7, *p* < 0.05), and multilesion (7.1% *vs* 40.5%, *p* < 0.05) than patients in the UCB group.

### 3.5. Surgical and Prognostic Outcome of Patients in the NPB Group

Of the patients with NPB, 10 underwent TURBT, and 4 underwent partial cystectomy (open, laparoscopic, or robot-assisted laparoscopic surgery). However, one of the patients who underwent TURBT required extra partial cystectomy for tumor residual. Moreover, 1 patient was pathologically misdiagnosed with muscle-invasive UBC after TURBT (performed at a municipal hospital). Although we performed salvage cystectomy on him after recurrence, the patient indirectly died of metastasis (radiotherapy-induced intestinal obstruction).

The mean follow-up time was 72.7 ± 32.3 months for patients with NPB. Except for the patient who indirectly died of metastasis, all patients with NPB survived without recurrence or metastasis.

## 4. Discussion

As our results have shown, misdiagnosis of NPB as UCB is widespread, which may result from tremendous amounts of pitfalls. Along with the rarity of NPB, the similarities in symptoms between NPB and UCB mostly result in frequent misdiagnosis [2]. Furthermore, normal catecholamine/metabolite levels and negative results in functional imaging [9, 10] exceedingly increase the difficulty in differentiating NPB from UCB. In addition, our findings suggest that NPB tends to have a similar size to UCB and commonly located adjacent to the mucosal layer (submucosal lesion), which are another 2 potential factors that may result in misdiagnosis.

However, NPBs and UCBs are fundamentally distinct in surgical approaches, adjuvant therapies, follow-up protocols, and expected prognoses. Thus, an accurate diagnosis is critical as misdiagnosis may result in severe consequences. In this study, we aimed to compare the differences in clinical characteristics and cystoscopic features between NPB and UCB.

Our findings demonstrated that patients with NPB significantly tend to be younger when compared with patients with UCB. However, the age of patients with NPB is extensively variant (see Table 1) and partly overlaps with that of patients with UCB. Thus, age can only provide a minor clue to the differential diagnosis. Unlike UCB, which occurs more frequently in males, sex difference in patients with NPB is not evident. The sex difference between NPB and UCB, to a certain extent, may be another clue to the differential diagnosis. Furthermore, our findings suggest that patients with NPB are less likely to present with hematuria.

Even though differences in age, sex, and hematuria between NPB and UCB can be redolent of the differential diagnosis, as the gold standard for diagnosing bladder tumors, cystoscopy is exceedingly essential for NPB. The reasonability of performing cystoscopy on a patient with paraganglioma of the bladder is controversial due to the potential risks of catecholamine crisis [11–16]. However, theoretically and empirically, cystoscopy is relatively safe for a patient with NPB on the condition of strictly excluding functional paragangliomas of the urinary bladder.

**Figure 1:**
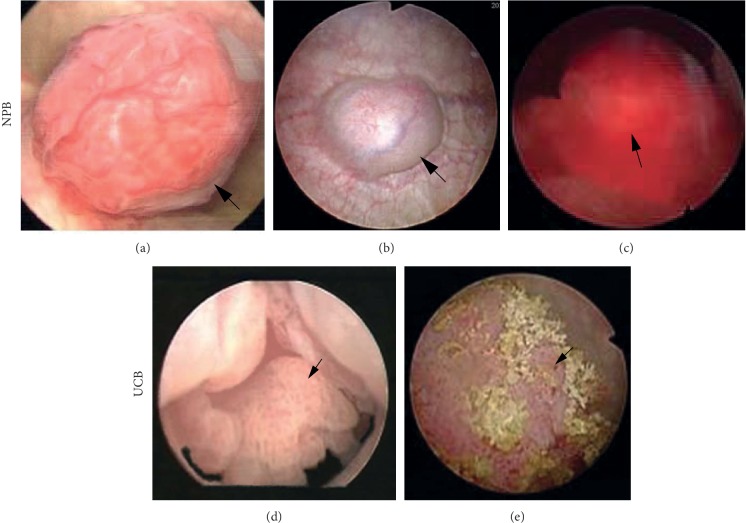
Cystoscopy of NPB and UCB. (a) A submucosal NPB with hypervascular spherical pedunculated solid mass covered with glossy mucosa. (b) An intramural NBP with hemispherical bulged mass with a broad base. (c) An intramural NPB with severe hemorrhage and obscure profile. (d) A papillary non-muscle-invasive UCB with pedicle. (e) A solid muscle-invasive UCB with hemorrhage, necrosis, and calcification. NPB = nonfunctional paraganglioma of the bladder; UCB = urothelial carcinoma of the bladder.

As the tumor originates from the chromaffin tissues embedded in the muscular layer or the lamina propria instead of the mucosal layer, NPB presents with predominantly different cystoscopic features from UCB. Cystoscopically, NPB often presents with a solitary hypervascularized solid mass covered with glossy mucosa (Figure 1(a)) or occasionally bleeding mucosa (Figure 1(c)). The mass barely floats with flushing fluid during cystoscopy. Necrosis and calcification on the surface of the lesion are infrequent. The lesion color is diverse (Figures 1(a)–1(c)), varying from pink (similar to the mucosa) to red and further to dark red, which may be related to the thickness of the covering tissue, abundance of the blood supply, and hemorrhage of the mucosa. The profile for the submucosal type of NPB is pronouncedly observable, either spherical or irregular and pedunculated or nonpedunculated. In contrast, the profile for the intramural type of NPB is usually hemispherical and characterized by bulged mass with a broad base (Figure 1(b)). However, its profile may not be as observable as that of submucosal NPB, but only the intravesical part of the mass is observable.

Cystoscopically, UCB frequently presents with hemorrhage, necrosis, calcification, and multilesion (Figures 1(d) and 1(e)). Most UCBs are non-muscle-invasive, presenting with papillary tumors (except for carcinoma in situ) and usually floating with flushing fluid. Muscle-invasive UCBs normally present with solid dark red or brown masses with conspicuous profiles and do not float with the flushing fluid.

In our study, the cystoscopic differences in hemorrhage, necrosis, calcification, pedunculation, hypervascularization, and lesion number between NPB and UCB were statistically significant. Namely, NPB tends to present with intact mucosa and hypervascularization but without pedunculation or multilesion. These findings are valuable and helpful indicators for the differential diagnosis from UCB.

**Figure 2:**
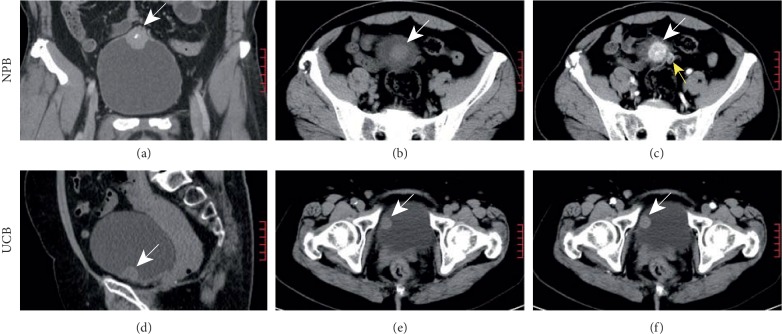
Computed tomography of NPB and UCB. (a–c) A solitary solid NPB with oval profile and intense enhancement, arteries can be observed (yellow arrow). (d–f) A papillary UCB with mild enhancement. NPB = nonfunctional paraganglioma of the bladder; UCB = urothelial carcinoma of the bladder.

On cystoscopy, most NPBs are readily differentiable from urothelial carcinoma, squamous cell carcinoma, and adenocarcinoma, according to the glossy and continuous mucosa. However, NPB occasionally presents with hemorrhage, necrosis, or both (Figure 1(c)), which may pose an obscure image and the potential misdiagnosis. Furthermore, hemorrhage denotes invasion of the mucosal layer and easiness to obtain specimen from the lesion. Consequently, for a rare entity, a biopsy is required and feasible to establish a preoperative diagnosis. In the case demonstrated in Figure 1(c), the biopsy played a critical role in correcting the previous misdiagnosis and avoiding the intended radical cystectomy (Figures 1(c) and 2(a)–2(c) from the same patient, who was suspected with muscle-invasive UBC. Eventually, robot-assisted partial cystectomy was performed after the biopsy). Moreover, a biopsy is necessary to the differential diagnosis of NPB from other nonepithelial tumors, e.g., sarcoma and leiomyoma [17].

Of note, biopsy should be taken deeply enough because the lesion is mainly located in the muscular layer or the lamina propria. A nondiagnostic or inconclusive biopsy is reported not rare [12, 13, 18]. If possible, hemostatic equipment should be prepared in case of massive hemorrhage given the abundant blood supply and required depth during the biopsy. Notably, functional paraganglioma of the bladder should be strictly excluded to avoid catecholamine crisis, e.g., hypertensive crisis [19], catecholamine cardiomyopathy, and arrhythmia.

On CT, NPB is characterized by a solid mass with round or oval profile, usually a solitary lesion (Figures 2(a)–2(c)). On enhanced CT, NPB presents with an intensely enhanced mass with abundant blood supply, and even arteries can be identified occasionally (Figure 2(c)). In contrast, most UCBs present with mild-to-moderate enhancement on CT (Figure 2(f)), and multilesion and calcification can be detected. Although all patients in the NPB group were misdiagnosed with bladder cancer before cystoscopy, their different CT characteristics from UCB may provide clues to the differential diagnosis.

Surgical tumor removal is the most effective treatment for functional paraganglioma of the bladder or NPB [20]. Partial cystectomy is assumed as a more reliable and preferred surgical approach than TURBT [20, 21]. However, to our best knowledge, no studies have evaluated the surgical approaches by different functional types of paragangliomas of the bladder. In the NPB group, 80.0% (8/10) of patients treated with TURBT showed no signs of tumor residual or recurrence. With minimal invasion, TURBT can be curable for some NPB cases. However, it should be based on strict selection, intraoperative/postoperative assessments, and regular follow-up. In our experience, when selecting TURBT, tumor size, anatomical type, breadth of the base, abundance of the blood supply, depth of the lesion, and signs of malignancy should be taken into account. Furthermore, 2 of our patients were successfully treated with diagnostic TURBT, which appears to be feasible.

**Figure 3:**
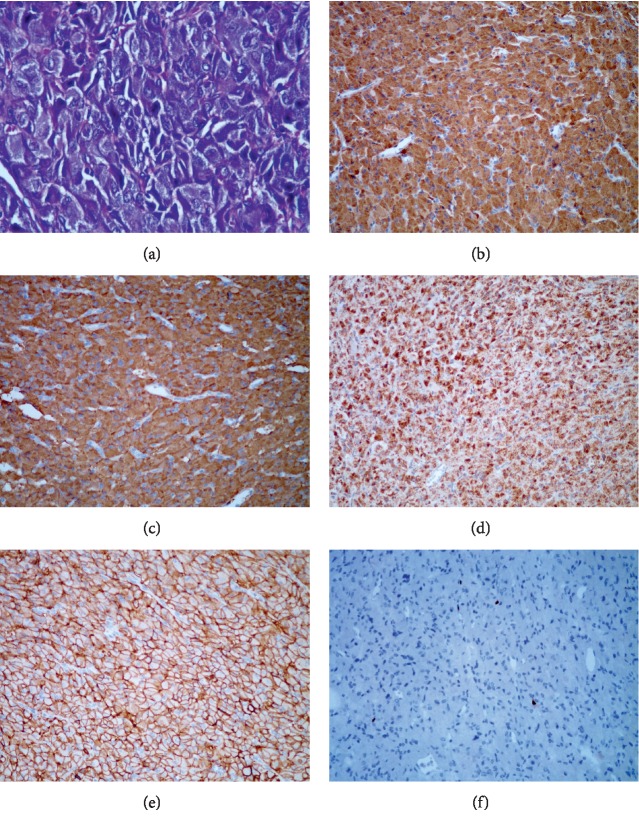
Histological and immunohistochemical examinations. (a) H&E staining (400X) showing zellabllen pattern and large nuclei with dark color. (b–f) Immunohistochemical staining (200X) shows positive expression of CgA, Syn, SDHB, CD56, ki67, respectively. H&E = haemotoxylin and eosin; CgA = chromogranin A; Syn = synapsin; SDHB = succinate dehydrogenase complex iron sulfur subunit B.

Notably, pathological examinations on the specimen from TURBT or biopsy may lead to misdiagnosis [6–8, 22]. One of our patients with NPB was pathologically misdiagnosed with UCB after TURBT. Unfortunately, the tumor was proved to be malignant eventually. The pathological misdiagnosis could have resulted in the subsequent metastasis and death. Therefore, careful histological and immunohistochemical examinations are required to avoid postoperative misdiagnosis (Figure 3).

Our study found significant differences in clinical characteristics and cystoscopic features between NPB and UCB. These findings may facilitate the differential diagnosis of NPB from UCB and potentially decrease the probability of misdiagnosis.

However, our study has limitations. First, the NPB group had both submucosal and intramural types, which had differences in cystoscopic features. The small sample sizes led to the invalidity of comparing them with UCB separately. Moreover, by random minimizing the UCB sample though deterministic and minimizes significant power losses, may not equally reflect the entire data pool. Second, all the crystals that adhered to the tumor surface were considered as calcification due to the difficulty in distinguishing stone fragments from calcification. Lastly, the length of the follow-up was relatively short to assess the prognosis of patients who underwent TURBT, given the potential of late recurrence [23].

## 5. Conclusions

Although NPB symptomatically mimics UCB, age, sex, and hematuria are possible clues to differentiating NPB from UCB. Compared with UCB, cystoscopically, NPB is more likely to present with hypervascularization but less likely to present with hemorrhage, necrosis, calcification, pedunculation, and multilesion. These cystoscopic features of NPB are the key to distinguishing it from UCB. Cystoscopic biopsy should be considered in the preoperative diagnosis of NPB.

## Figures and Tables

**Table 1:** Characteristics of patients with nonfunctional paraganglioma of the urinary bladder.

No.	Age (year)	Sex	Hematuria	Anatomical type	Diameter (cm)
1	27	Male	No	Submucosal	2.4
2	29	Male	No	Submucosal	1.0
3	30	Male	Yes	Submucosal	0.8
4	31	Male	Yes	Submucosal	5.0
5	34	Female	Yes	Intramural	4.0
6	37	Male	Yes	Intramural	2.7
7	39	Male	No	Submucosal	1.8
8	39	Female	No	Submucosal	2.2
9	44	Female	No	Submucosal	1.1
10	50	Female	No	Intramural	1.0
11	55	Female	No	Submucosal	1.0
12	58	Female	No	Submucosal	0.7
13	60	Female	Yes	Submucosal	4.0
14	71	Male	No	Submucosal	1.9

**Table 2:** Differences in general clinical characteristics between the 2 groups.

Variable	NPB	UCB	*p* value	Statistical method
Age	43.1 ± 13.6	59.8 ± 12.0	<0.001	Student's *t*-test
Sex (%)				
Male	7 (50)	34 (81.0)	0.037	Fisher's exact test
Female	7 (50)	8 (19.0)		
Hematuria (%)				
Yes	5 (35.7)	35 (83.3)	0.001	Fisher's exact test
No	9 (64.3)	7 (16.7)		
Maxi-diameter	1.9 (IQR: 1.0–2.6)	2.0 (IQR: 1.0–3.0)	0.609	Wilcoxon rank-sum test

NPB = nonfunctional paraganglioma of the bladder; UCB = urothelial carcinoma of the bladder; and IQR = interquartile range.

**Table 3:** Differences in cystoscopic features between the 2 groups.

Variable	NPB	UCB	*p* value	Statistical method
Hypervascularization (%)				
Yes	10 (71.4)	9 (21.4)	0.001	Fisher's exact test
No	4 (28.6)	33 (78.6)		

Hemorrhage (%)				
Yes	4 (28.6)	30 (71.4)	0.004 (*χ*^2^ = 8.1)	Chi-square test
No	10 (71.4)	12 (28.6)		

Necrosis (%)				
Yes	1 (7.1)	16 (38.1)	0.042	Fisher's exact test
No	13 (92.9)	26 (61.9)		

Calcification (%)				
Yes	0	13 (31.0)	0.025	Fisher's exact test
No	14 (100.0)	29 (69.0)		

Pedunculation (%)				
Yes	5 (35.7)	30 (71.4)	0.017 (*χ*^2^ = 5.7)	Chi-square test
No	9 (64.3)	12 (28.6)		

Multilesion (%)				
Yes	1 (7.1)	17 (40.5)	0.023	Fisher's exact test
No	13 (92.9)	25 (59.5)		

NPB = nonfunctional paraganglioma of the bladder; UCB = urothelial carcinoma of the bladder.

## Data Availability

All data and materials can be obtained by e-mail to the corresponding author.

## Additional Points

*Clinical Practice Points*. (i) Symptomatically, NPB largely mimics UCB, which exceedingly increases the difficulty in the differential diagnosis. (ii) Age, sex, hematuria, and imaging characteristics are potential clues to differentiating NPB from UCB. (iii) On cystoscopy, NPB tends to present with hypervascularization but without hemorrhage, necrosis, calcification, pedunculation, and multilesion. (iv) A biopsy is recommended for preoperative diagnosis of NPB.
